
JOURNAL INFORMATION
==============================
NLM Title Abbreviation: Med Chir Rev
NLM Journal ID: 0034617

The Medico-Chirurgical Review

Publisher: Biomedical Journal Digitization Project

ARTICLE INFORMATION
==============================
PMCID: PMC4966194
PMID: 29917616
Article version: 1
Subjects: Reviews

On Spasmodic Asthma (Eclectic)

Print publication date: 1828 Jul 1
Volume: 9
Issue: 17
First page: 141
Last page: 144
1828 Jun 21
License: This work is free of known copyright restrictions. For more information, please see PMC Back Issue Digitization.
License URL: https://creativecommons.org/publicdomain/mark/1.0/

==============================

from the facts we have seen, and the observations we have made, that fever presents as many varieties of symptoms as there are varieties of causes, constitutions, and circumstances, and that it may appear under every grade of inveteracy, from the most putrid type, where life is seen sinking in a mass of rottenness, up to the most intense phlogistic character, where the lancet must be handled and handled freely; we have been compelled to impugn many positions that were perfectly consistent with the author's leading doctrine. Fever must not only be seen to be described, but it must be seen under every changing form which changing circumstances can attach to it. In one epidemic its prominent feature its debility, in another it is strength. In one, enteritic symptoms will be found to predominate, in another, disorder of the head. In one constitution every important organ will successively feel the influence of its power, in another, it distributes its excitement in equal proportion throughout the body, passing along its stages without characterizing its attacks or stopping its progress, by any local affection, or leading symptom.

All these varieties must be seen and studied, and every modification of feature must be marked and noted, before we can, either with safety or success, sit down to delineate a faithful portrait. Had Dr. Burne confined himself to a certain epidemic, or to a certain combination of febrile phenomena entitled to the epithet he employs, his work had not occasionally sacrificed its fidelity to Nature for the sake of consistence, and the claims which it certainly has upon public patronage, had not been deteriorated by a comparison of the limited applicability of the principles it inculcates with the extensive magnitude of the subject which it treats.

---

### XIII.

### ON ASTHMA.*

SPASMODIC or periodical asthma has been confounded, even by Hoffman, Floyer, and many others, with dyspnœa, or difficulty of breathing, a phenomenon produced by very many organic affections, as chronic catarrh, hepatization of the lungs, hydrothorax, and the various diseases of the heart. It is not difficult to see the mischief which must have resulted from this confounding of affections of the most different kind. But the modern school of Continental pathologists have run into an opposite extreme, and have denied the possibility of a purely spasmodic or periodical asthma—because they have found the same symptoms accompanying organic diseases. The indefatigable Laennec has taken

* M. Ferrus—Laennec—Lullier-Winslow—Forbes—Rostan, &c. &c. &c.

great pains to disentangle the question of the difficulties with which it has long been surrounded, and as his work is not yet sufficiently diffused through the profession, we shall lay a brief exposé of his sentiments before our readers in this article.

With the view of shewing that spasmodic asthma may exist, without any violence to physiology, he endeavours to prove that there are circular fibres around the bronchial ramifications, beginning at the point where the cartilaginous circles terminate. Analogy, he thinks, must lead us to admit the existence of the same kind of muscular fibres in the smaller branches, and even in the air-cells.

"Taking this view of the subject, it is very conceivable that the spasmodic contraction of these fibres may be carried the length of obstructing the air passages to such a degree as to prevent the transmission of air to a great portion of the lungs. For these reasons, we cannot regard the tonic spasm of the bronchia, or even perhaps of the air cells, as impossible; since every muscle is susceptible of spasm. Besides it is by no means demonstrated, that muscular fibre is the only contractile tissue; indeed, the contrary is proved by the fact, that animals of almost a mucilaginous consistence, are capable of evident contraction." 408.

Laennec is convinced that the lungs are possessed of an active power of expansion, as well as contraction.

"If (says he) we lay open one side of the chest of a living dog, we find the lung at first reduced to one fourth of its former dimensions; but even in this state we observe it swelling and contracting with an alternate motion. This fact was noticed by M. Roux (*Melanges de Chir. &c.* p. 87) who further remarks, that we cannot account for the escape of a portion of lung in the case of a wound of the chest, but by an active expansion of the viscus itself." 409.

The protruded portion has been observed to be dilated during inspiration, a result which, he thinks, could not be occasioned by atmospheric pressure. Many other reasons are adduced by our author for this inherent power of expansion in the pulmonary tissue; but we confess that to us they appear rather unsatisfactory—and moreover unnecessary. An attack of purely spasmodic asthma is rarely fatal—hardly ever so without giving rise to congestions of blood and other *consequences* of disordered respiration, which, by modern pathologists, are looked on in the light of *causes* of the asthmatic paroxysm.

"I have met with many cases in which it was impossible, after the most minute research, to find any organic lesion whatsoever, to which the asthma could be attributed. An instance of the same kind is given by M. Andral (Cl. Med. t. II. ob. XX.) in the case of a fatal suffocation supervening to the suppression of a discharge from an ulcerated leg. The lungs in this case were sound, except that there existed in the left lower lobe a small hepatized point, of less extent than the tenth part of the lobe (*pneumonia of the dying*, according to all appearances.) The heart and the other organs were equally sound. M. Guersent has likewise seen two children die, after a few days, of a remitting dyspnœa, attended with dry cough and precordial anxiety, in whose bodies no obvious lesion could be found after death. (Dict. de Med. t. III. p. 126.) I am convinced that in the greater number of asthmatic cases, depending on dry catarrh and pulmonary emphysema, the asthmatic paroxysm can be induced equally by the supervention of a fresh catarrh (latent or manifest), and by a deranged state of the nervous influence, occasioning pulmonary spasm or an increase of the necessity of respiration, and sometimes by both causes at once. In fact, I believe there are few cases of asthma owing to any one of these causes; and in old men more particularly, I imagine that several frequently conspire to produce the result. Of this kind are, debility, the ossification of the cartilages and immobility of the ribs, rheumatism affecting the walls of the chest, and perhaps also the tenuity of the air cells and of all the pulmonary vessels, in advanced life. With the exception of the different kinds of catarrh, the occasional causes of attacks of asthma and dyspnœa are almost always of a kind to give occasion to an immediate and evident disturbance of the nervous influence. Of this kind are, strong mental emotion; venereal excesses; the influence of light or darkness; retrocession of gout (an affection which, from its mobility and various effects, can only be considered as a nervous affection); certain odours, such as those of tuberose, heliotrope, stored apples, &c.; changes of the atmospheric electricity, and other less appreciable conditions of the atmosphere. We thus find that the greater number of asthmatic patients cannot remain with impunity in a low close apartment, although containing much more air than they could consume in twenty-four hours, and although it is constantly but insensibly renewed by the doors and chimneys. Some cannot bear, without experiencing a feeling of suffocation, that any person should go before them, or that any thing should be brought close to them; while others, on the contrary, are never more subject to dyspnœa than when in the midst of a vast plain. The following fact, communicated to me by one of my colleagues, affords a curious example of a nervous affection of a similar kind, in a person not subject to asthma. A man forty years of age, slightly hypochondriacal but otherwise in good health, wished to go on horseback to pay a visit some leagues distant from his house. As soon as he left the town where he resided, which is situated in an extensive plain, he felt an immediate oppression on the chest from the impression of the country air. He took no notice of this at first; but the dyspnœa having greatly increased, and being now attended by a sense of faintness, he determined to return. He had scarcely turned his horse, when he found himself better; and in a few minutes he recovered both his breath and his strength. Not suspecting any relation between this momentary uneasiness and his journey, he once more attempted to advance, and was again soon attacked with the dyspnœa and faintness. On turning towards the town these passed off. After having made repeated attempts to proceed, and always with the same result, he finally returned, and in just as good health as when he set out." 414.

*Treatment.* Since the periodical asthma commonly depends on the re-union of several organic and nervous affections, it is necessary to investigate each case with accuracy, in order to get at the elements of the disease. Thus, in many cases, it will be found to depend on catarrh, latent or manifest, and it is in such cases, that emetics, alkaline salts, squills, ipecacuan, have been found so useful. For the nervous part of the complaint, a great number of remedies have been recommended; but nothing is more variable than their effects. Some will succeed in one person and fail in another. Even in the same individual, the same medicine will produce different effects at different periods. Narcotics—quiet—darkness, have generally a good effect. Narcotics may act not merely by lessening the necessity of respiration, but also by overcoming the spasm of the lungs; "and we ought, therefore, to have recourse to them in every case where exploration of the chest enables us to detect either of these changes in the condition of the nervous influence." Opium and colchicum we have found to be the most powerful means of shortening and mitigating the paroxysm. Where the asthma takes a decidedly periodical character, with somewhat regular intervals, the tonic class of remedies is indicated, as iron, arsenic, and bark. M. Laennec has employed the carbonate of iron with much success, whether the asthma depended on dry catarrh or was purely nervous. "Whatever be the occasional causes, or the elements of the asthma, we must never omit blood-letting, whenever the lividity of the countenance, the strength of the patient's constitution, or the over-action of the heart, indicate pulmonary congestion; but we must be careful not to abuse this practice, which, in general, only produces a temporary advantage." We shall introduce the following short passage from one of Dr. Forbes's notes.

"Among the remedies best deserving notice in asthma, I would mention, a mild and spare diet, residence in a more temperate climate, and warm bathing. The first of these measures will be found very beneficial in cases complicated with gastric irritation; the two last are especially indicated in that class of cases which date from the disappearance of cutaneous eruptions under the use of powerful external applications. This method of cure is, I am convinced, the fruitful source of many internal irritations and inflammations, and, among others, of bronchitis and asthma. Although the doctrine of repulsion may be deemed by some theorists somewhat obsolete, I feel assured that its truth will be assented to by most observant practitioners of experience. I therefore consider it my duty to caution the student against a practice, which accords too well with the energetic empiricism so much in favour in this country, not to be readily adopted from analogy, even if not inculcated by positive precept." 419.

As asthma seldom continues long without inducing some changes in the lungs or heart, even if it has been purely spasmodic at the beginning, so we should not regard the disease so lightly as some have done. We have known it argued at insurance offices, that people who had spasmodic asthma for many years, were good and unexceptionable lives. We are not of this opinion. Because we see many asthmatics live to a good old age, we are not thence to conclude that no others have fallen victims to the malady. We see and remark the living:—we soon forget the dead! In all cases, we should institute a rigorous inquiry into the condition of the heart and lungs, in order to ascertain whether they are the seat of lesions, whether primary or secondary. When these lesions are detected—and they will too often be detected if carefully sought for, then our chief object should be to obviate their tendency, and prevent their aggravating the spasmodic disease.

P. S. In the present Number of this Journal, p. 208, will be found an account of the lobelia inflata, as a specific for spasmodic asthma. Mr. Snowden, chemist, in the Haymarket, has procured some of the plant, and is preparing a tincture according to the formula given. We recommend a trial of this medicine.—*Ed.*
